# Low expression of CHRDL1 and SPARCL1 predicts poor prognosis of lung adenocarcinoma based on comprehensive analysis and immunohistochemical validation

**DOI:** 10.1186/s12935-021-01933-9

**Published:** 2021-05-12

**Authors:** Huan Deng, Qingqing Hang, Dijian Shen, Yibi Zhang, Ming Chen

**Affiliations:** 1 College of Life Sciences, University of the Chinese Academy of Sciences, Beijing, 100049 China; 2 Department of Radiation Oncology, Cancer Hospital of the University of Chinese Academy of Sciences (Zhejiang Cancer Hospital), Hangzhou, 310022 China; 3 Institute of Cancer Research and Basic Medical (IBMC), Chinese Academy of Sciences, Hangzhou, 310022 China; 4 Department of Radiation Oncology, Zhejiang Key Laboratory of Radiation Oncology, Zhejiang Cancer Hospital, Hangzhou, 310022 China; 5 Zhejiang Chinese Medicinal University, Hangzhou, 310022 China; 6 Jiangxi Medical College, Nanchang University, Nanchang, 331800 China

**Keywords:** Lung adenocarcinoma, Weighted gene coexpression network analysis, Differential coexpression genes, Protein–protein interaction network, Survival analysis

## Abstract

**Purpose:**

Exploring the molecular mechanisms of lung adenocarcinoma (LUAD) is beneficial for developing new therapeutic strategies and predicting prognosis. This study was performed to select core genes related to LUAD and to analyze their prognostic value.

**Methods:**

Microarray datasets from the GEO (GSE75037) and TCGA-LUAD datasets were analyzed to identify differentially coexpressed genes in LUAD using weighted gene coexpression network analysis (WGCNA) and differential gene expression analysis. Functional enrichment analysis was conducted, and a protein–protein interaction (PPI) network was established. Subsequently, hub genes were identified using the CytoHubba plug-in. Overall survival (OS) analyses of hub genes were performed. The Clinical Proteomic Tumor Analysis Consortium (CPTAC) and the Human Protein Atlas (THPA) databases were used to validate our findings. Gene set enrichment analysis (GSEA) of survival-related hub genes were conducted. Immunohistochemistry (IHC) was carried out to validate our findings.

**Results:**

We identified 486 differentially coexpressed genes. Functional enrichment analysis suggested these genes were primarily enriched in the regulation of epithelial cell proliferation, collagen-containing extracellular matrix, transforming growth factor beta binding, and signaling pathways regulating the pluripotency of stem cells. Ten hub genes were detected using the maximal clique centrality (MCC) algorithm, and four genes were closely associated with OS. The CPTAC and THPA databases revealed that CHRDL1 and SPARCL1 were downregulated at the mRNA and protein expression levels in LUAD, whereas SPP1 was upregulated. GSEA demonstrated that DNA-dependent DNA replication and catalytic activity acting on RNA were correlated with CHRDL1 and SPARCL1 expression, respectively. The IHC results suggested that CHRDL1 and SPARCL1 were significantly downregulated in LUAD.

**Conclusions:**

Our study revealed that survival-related hub genes closely correlated with the initiation and progression of LUAD. Furthermore, CHRDL1 and SPARCL1 are potential therapeutic and prognostic indicators of LUAD.

**Supplementary Information:**

The online version contains supplementary material available at 10.1186/s12935-021-01933-9.

## Introduction

As one of common cancers, lung carcinoma was estimated to have caused disease in 235,760 patients and 131,880 deaths in 2021, resulting in a tremendous burden to our society [1]. Patients with NSCLC accounted for nearly 85% of all patients with lung cancer, and the most prevalent pathological pattern of NSCLC was lung adenocarcinoma (LUAD) [2]. In recent decades, many researchers have concentrated on studying the potential biological and molecular mechanisms of lung cancer, and the molecular mechanisms are gradually being understood [3]. It was recognized that identifying key molecular abnormalities would promote the rapid development of precision medicine, and more effective strategies will be identified for the diagnosis, treatment and prognosis of LUAD in the near future [4]. Currently, it is essential for us to identify core genes associated with the carcinogenesis and development of LUAD.

With the rapid advancement of genomic technology, bioinformatics analyses have been widely used in the analysis of microarray datasets to further study the potential molecular mechanisms of cancers and to identify tumor-specific indicators [5]. Weighed gene coexpression network analysis (WGCNA) is one of these significant algorithms that provides a better understanding of gene coexpression networks and gene functions [6]. WGCNA can detect modules of highly correlated genes among samples to relate modules to external sample traits, providing valuable insights into predicting possible functions of coexpressed genes [7]. Moreover, differential gene expression analysis is usually used in transcriptomics datasets to study underlying biological and molecular mechanisms and to identify quantitative differences in the expression level of the gene between different groups [8].

To improve the discriminating ability of highly related genes, the two methods mentioned above were applied in our study. First, mRNA expression datasets of LUAD were obtained from Gene Expression Omnibus (GEO) and The Cancer Genome Atlas database (TCGA). Second, WGCNA and differential gene expression analysis were used to identify common differential coexpression genes. Then, we carried out functional enrichment analysis, protein–protein interaction (PPI) analysis, and overall survival analysis to identify potential biomarkers correlated with the occurrence and progression of LUAD. Next, the expression patterns of hub genes at the mRNA and protein levels were verified through GSE19188 from GEO, Clinical Proteomic Tumor Analysis Consortium (CPTAC) and the Human Protein Atlas (HPA). Furthermore, we conducted gene set enrichment analyses (GSEA) of survival-related hub genes using the TCGA-LUAD dataset. Ultimately, we carried out immunohistochemistry (IHC) analysis of survival-related hub genes for further validation.

## Materials

**Fig. 1:**
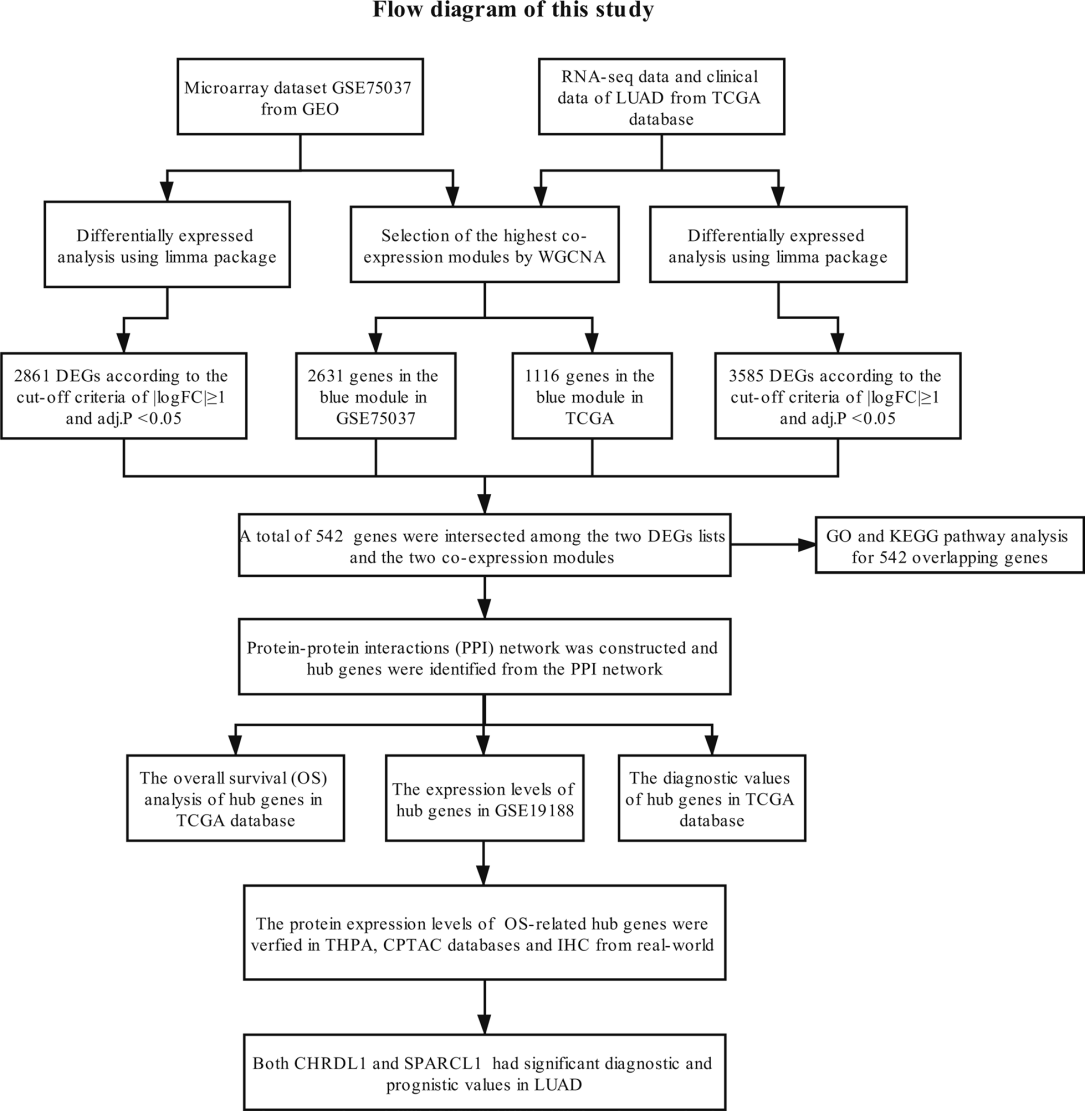
Study design and workflow of our study

Figure 1 reveals the detailed processes of data download, hub gene identification and external validation. Every step is illustrated in the following subsection.

### Datasets from GEO and TCGA

The mRNA expression datasets of LUAD were acquired from the GEO and TCGA databases. First, one microarray dataset (GSE75037) was selected from GEO, and this dataset included 83 LUAD tissues and 83 matched nonmalignant adjacent tissues from LUAD patients. GSE75037 was based on the GPL6884 Illumina HumanWG-6 v3.0 expression beadchip. Based on the manufacturer-provided annotation file, probes would be transformed to corresponding gene symbol, probe sets without gene symbol would be removed, and duplicated probes for the same gene would be averaged. Consequently, a total of 25,428 genes were acquired for the next analysis. Second, the mRNA expression data and corresponding clinical information of LUAD were acquired from TCGA. 594 LUAD samples were downloaded, consisting of 535 LUAD and 59 normal lung tissues, and RNAseq data about fragments per kilobase per million (FPKM) on 19,145 genes were obtained. Then, FPKM data were transformed to transcript per million (TPM) data for the next analysis. Based on the Illumina HiSeq 2000 platform, all the data were generated and annotated to a reference transcript set of Human hg38 gene standard tracks. The edgeR package tutorial showed that genes with low read counts were probably meaningless for the next analysis [9]. Therefore, genes with TPM < 1 were removed from our study. As a result, 15,142 genes were obtained for the subsequent analysis.

### Identification of core coexpression modules through WGCNA

**Fig. 2:**
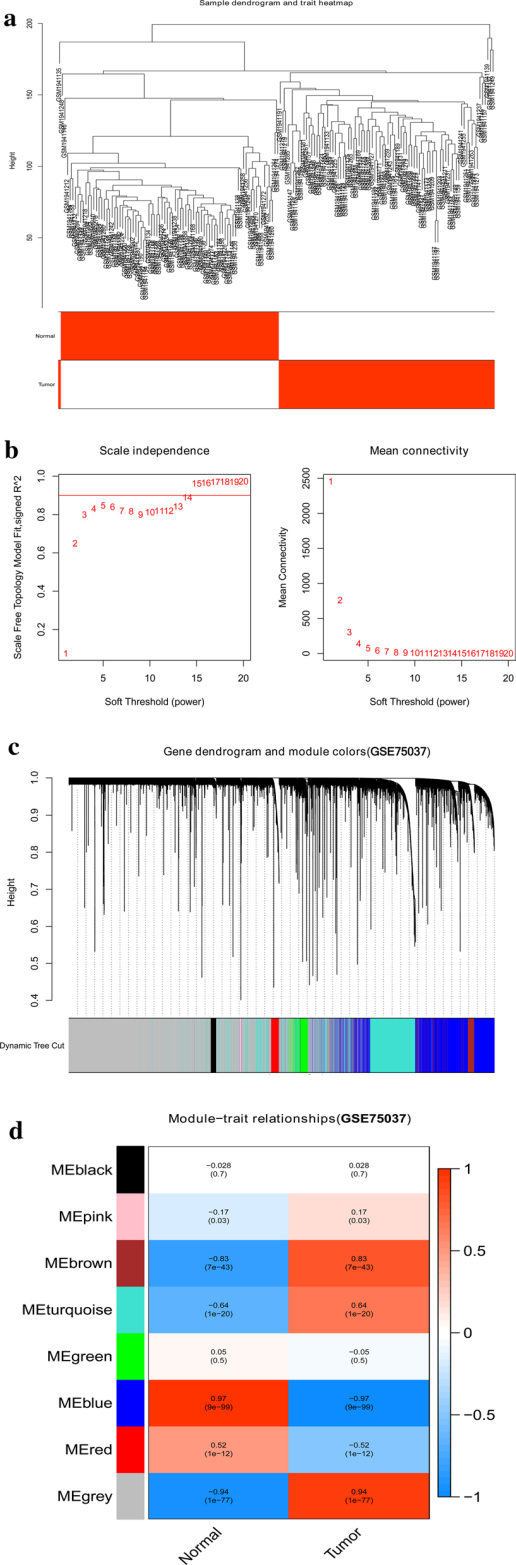
Identification of modules correlated with the clinical traits in GSE75037. **a** Sample dendrogram and trait heatmap. **b** Scale independence and Mean connectivity. **c** The Cluster dendrogram of co-expression network modules is ordered by a hierarchical clustering of genes based on the 1-TOM matrix. Different colors represent different modules. **d** Module-trait relationships. Each row represents a color module and every column represents a clinical trait (normal and tumor). Each cell contains the corresponding correlation and P-value

**Fig. 3:**
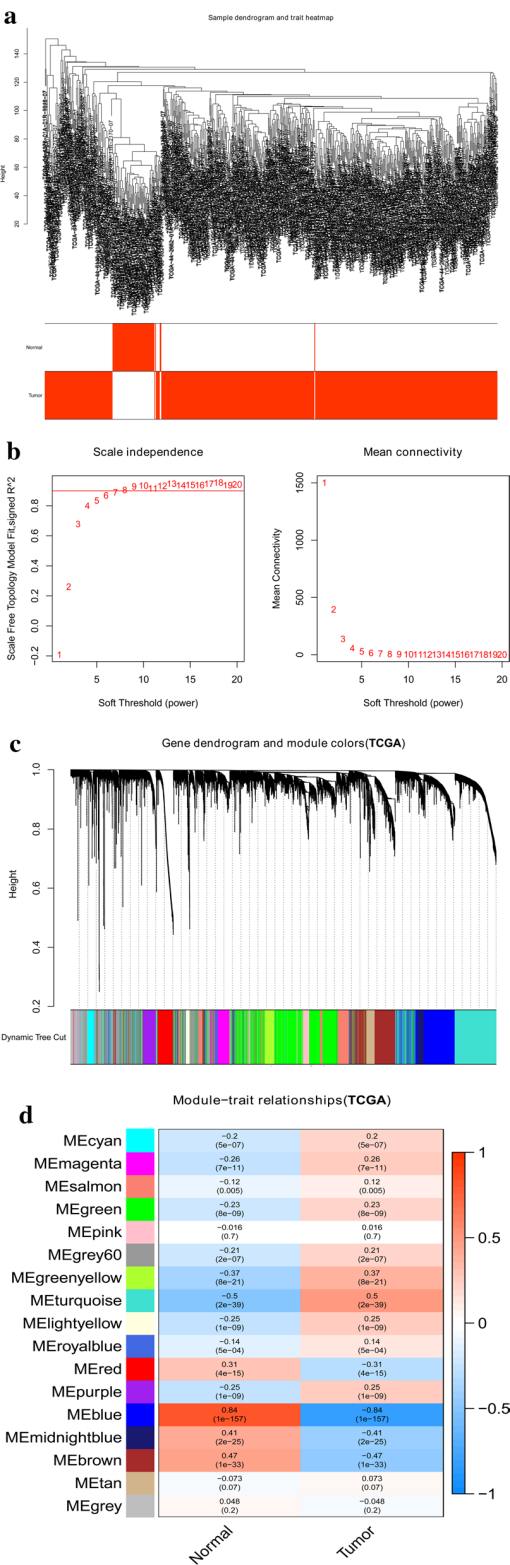
Identification of modules correlated with the clinical traits in TCGA-LUAD dataset. **a** Sample dendrogram and trait heatmap. **b** Scale independence and mean connectivity. **c** The Cluster dendrogram of co-expression network modules is ordered by a hierarchical clustering of genes based on the 1-TOM matrix. Different colors represent different modules. **d** Module-trait relationships. Each row represents a color module and every column represents a clinical trait (normal and tumor). Each cell contains the corresponding correlation and P-value

We constructed gene coexpression networks of the GSE75037 and mRNA expression profiles of the TCGA-LUAD dataset through the WGCNA package. WGCNA was used to identify highly related genes and aggregate these genes into the same genetic module related to external sample traits. To construct a scale-free network, soft powers β = 14 (Fig. 2b) and 6 (Fig. 3b) were used for GSE75037 and mRNA expression profiles of TCGA-LUAD, respectively. Then, the adjacency matrix was generated using the following formula: aij =|Sij|^β^ (aij: adjacency matrix between gene i and gene j, Sij: similarity matrix, which is determined by Pearson correlation of all gene pairs, and β: softpower value); then, the matrix was converted to a topological overlap matrix (TOM) and the corresponding dissimilarity (1-TOM). Subsequently, we established a hierarchical clustering dendrogram of the 1-TOM matrix to aggregate genes with similar expression into a coexpression module. The module-trait relations between modules and clinical trait information were explored for further identification of functional modules in the coexpression network. Thus, the module with the highest correlation coefficient was selected as the candidate module related to clinical traits, which was used for our next analysis.

### Selection of differential coexpression genes

The limma and edgeR packages were applied to perform differential expression analysis of microarray and RNA-Sequencing datasets, respectively [9, 10]. To select differentially expressed genes (DEGs) between LUAD tissues and nonmalignant tissues, we respectively used the limma and edgeR packages in the selection of DEGs from the GSE75037 and TCGA-LUAD datasets. To reduce the false discovery rate (FDR), the p-value was adjusted using the Benjamini–Hochberg method. The selection criteria for DEGs were set as |logFC| > 1 and adj. P < 0.05. To better discriminate highly related genes, we determined the intersection of genes among two lists of DEGs and two lists of coexpressed genes from the two coexpression networks, which were applied to identify candidate prognostic indicators of LUAD.

### Functional enrichment analysis

To analyze the biological functions of differentially coexpressed genes, GO and KEGG pathway analysis was conducted with the clusterProfiler [11] and GOplot packages. GO and KEGG are essential bioinformatics tools, which annotates gene and analyzes the biological process of genes [12]. P < 0.05 was considered statistically significant.

### PPI network construction and hub gene selection

The PPI network of differentially coexpressed genes was established with the Search Tool for the Retrieval of Interacting Genes (STRING) [13]. Cytoscape (version 3.7.2) was used to build a visual network of molecular interactions with a combined score > 0.6 [14]. The Molecular Complex Detection (MCODE) plugin in Cytoscape was used to detect highly correlated modules in PPI network [15]. The most significant gene module was visualized and graphically displayed using the MCODE plug-in. The criteria of selection were as follows: MCODE score > 5, node score cutoff = 0.2, degree cutoff = 2, k-score = 2, and max depth = 100. Additionally, the maximal clique centrality (MCC) algorithm was recognized to be the most useful approach to detect hub nodes from the PPI network [16]. We calculated the MCC score of every gene in the PPI network using the CytoHubba plug-in. Differentially co-expressed genes with the top ten highest MCC scores were believed to be hub genes. These hub genes were also visualized using the CytoHubba plug-in.

### Prognostic roles and relationship with pathological stages of hub genes

To explore the prognostic values of hub genes in LUAD, Kaplan–Meier univariate survival analysis was conducted through the survival package. Only patients with complete follow-up information were included for overall survival (OS) analysis, and we classified these patients into two cohorts in accordance with the median expression level of hub genes. Log-rank p < 0.05 was considered statistically significant. Additionally, we explored the relationship between their expression patterns and pathological stages among LUAD.

### External validation of GEO, CPTAC and THPA databases

To improve the reliability of our analysis, the GEO, CPTAC and THPA databases were used to validate the expression patterns of survival-related hub genes between LUAD and nonmalignant samples. To systematically analyze the mRNA expression patterns of survival-related hub genes between LUAD and nonmalignant samples, meta-analyses were carried out using relevant data from GEO. The search strategy and selection criteria for the included datasets in the GEO database are shown (Additional file 3: Table S1). Additionally, their protein expression patterns between LUAD and nonmalignant samples were explored using IHC outcomes from HPA [17] and quantitative comparison from CPTAC database [18].

### GSEA of survival-related hub genes

We divided these samples into two cohorts according to the median expression values of hub genes associated with OS. The effect of the expression of hub genes on multiple gene sets was analyzed for related GO enrichment analysis using c5.all.v7.2.symbols.gmt [gene ontology] [19]. The permutation of each analysis was set to 1000 times. |Normalized enrichment score (NES)| > 1, NOM p-value < 0.05 and FDR q-value < 0.25 were considered significant differences.

### Immunohistochemical verification

Twenty pairs of LUAD and normal tissues had been collected in Zhejiang Cancer Hospital (Zhejiang, China) from 2017 to 2021 (Additional file 4: Table S2). IHC was approved by the Medical Ethics Committee of Zhejiang Cancer Hospital (IRB-2020-817). These tissue samples were frozen in liquid nitrogen for next analysis. After epitope retrieval, hydrogen peroxide treatment and nonspecific antigen blocking, we incubated the sections of these tissues via deparaffinization and dehydration using anti-CHRDL1 (dilution: 1:500, PA5-78591, Thermo, USA) and anti-SPARCL1 antibodies (dilution: 1:1000, ab255597, Abcam, UK) overnight at 4 °C. Afterward, we incubated these sections using secondary antibodies (dilution: 1:200, ab150115, Abcam, UK). All sections were covered with Fluoroshield containing 4′,6-diamidino-2-phenylindole (DAPI, Abcam) for 10 min to identify nuclei, and we detected the signal using the DAB staining kit (Vector Laboratories, USA). The intensity was denoted as 0 (negative), 1+ (weakly positive), 2+ (moderately positive), and 3+ (strongly positive). H-score values (range 0–300) were calculated according to the following formula: [(% cells with an intensity of 1+) + 2 × (% cells with an intensity of 2+) + 3 × (% cells with an intensity of 3+)]. Two pathologists independently estimated scores of all sections, and mean scores were calculated as H-score values. IHC was independently repeated in triplicate, and student’s t-test was applied for comparisons between LUAD and normal lung tissue groups.

## Results

### Identification of core coexpression modules through WGCNA

To detect the functional modules in LUAD, we established two gene coexpression networks using the GSE75037 and TCGA-LUAD datasets through the WGCNA package in R software. We found eight modules (Fig. 2c) and 17 modules (Fig. 3c) in the GSE75037 and TCGA-LUAD datasets, respectively (one color represents one module). Next, the heatmaps explored the relationship between modules and two clinical traits (normal and LUAD) in the GSE75037 (Fig. 2d) and TCGA-LUAD datasets (Fig. 3d), suggesting that the blue module in GSE75037 and the blue module TCGA-LUAD dataset were closely associated with normal tissues (blue module in GSE75037: r = 0.97, p = 9e−99; blue module in TCGA-LUAD dataset: r = 0.84, p = 1e−157).

### The intersection of DEGs and coexpression genes

**Fig. 4:**
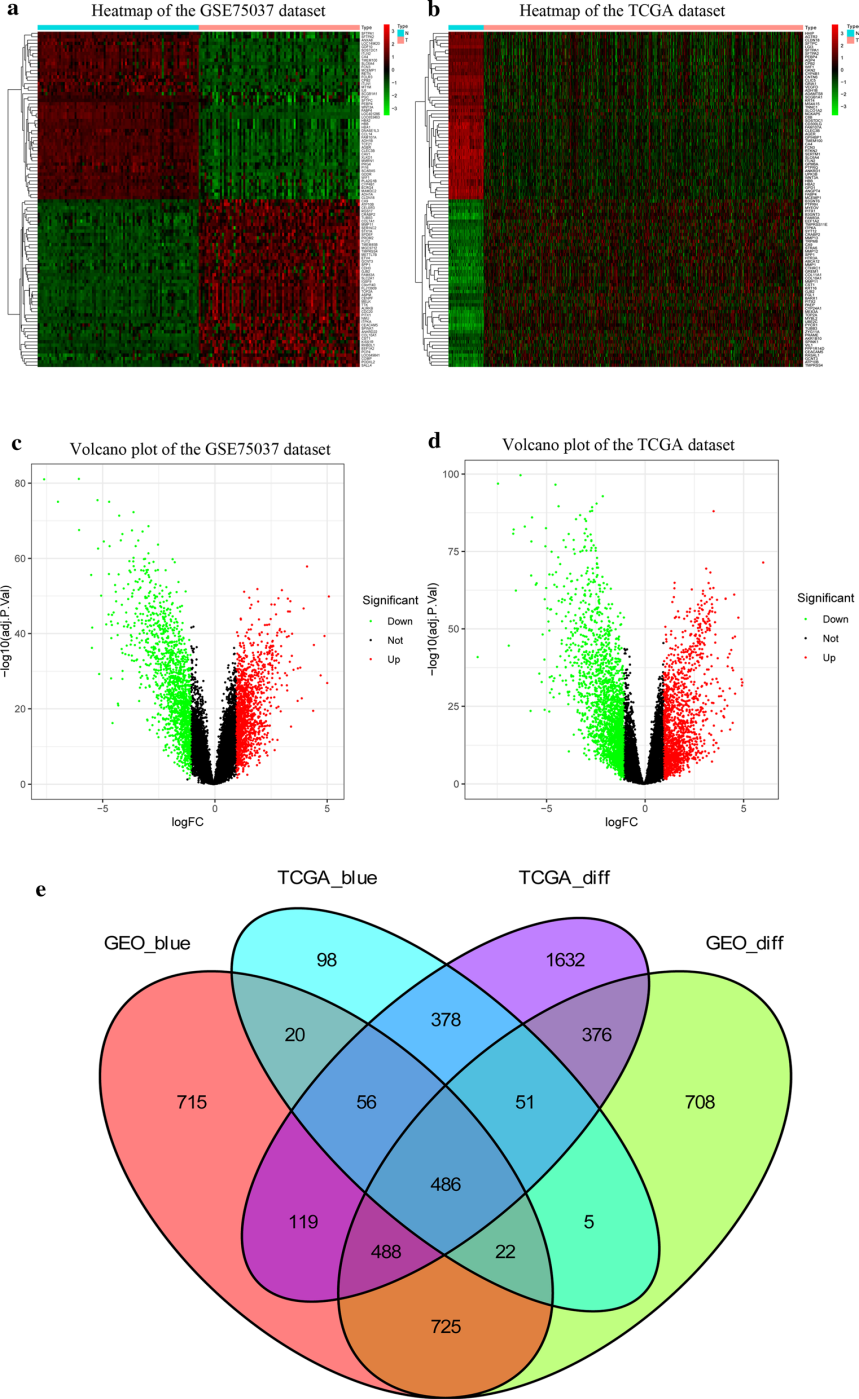
Identification of differentially expressed genes (DEGs) among GSE75037 TCGA-LUAD dataset with the cut-off criteria of |logFC| > 1 and adj.P < 0.05. **a** Heatmap of 50 upregulated and 50 downregulated DEGs of GSE75037. **b** Heatmap of 50 upregulated and 50 downregulated DEGs of TCGA-LUAD dataset. **c** Volcano plot of DEGs in GSE75037. **d** Volcano plot of DEGs in TCGA-LUAD dataset. **e** The Venn diagram of genes among the two DEG lists and the two lists of co-expression genes. In total, 486 overlapping differential co-expression genes are found

The heatmaps showed the expression patterns of 50 upregulated and 50 downregulated genes in the GSE75037 (Fig. 4a) and TCGA-LUAD datasets (Fig. 4b). The volcano plots illustrated that 2861 DEGs in GSE75037 (Fig. 4c) and 3585 DEGs in TCGA-LUAD dataset (Fig. 4d) were significantly dysregulated. Figure 4e clearly demonstrates that the intersection of two lists of DEGs (Additional file 5: Table S3; Additional file 6: Table S4) and two lists of coexpressed genes (Additional file 7: Table S5; Additional file 8: Table S6) contained 486 genes, which were used for the subsequent analysis (Additional file 9: Table S7).

### Functional enrichment analysis of differentially coexpressed genes

**Fig. 5:**
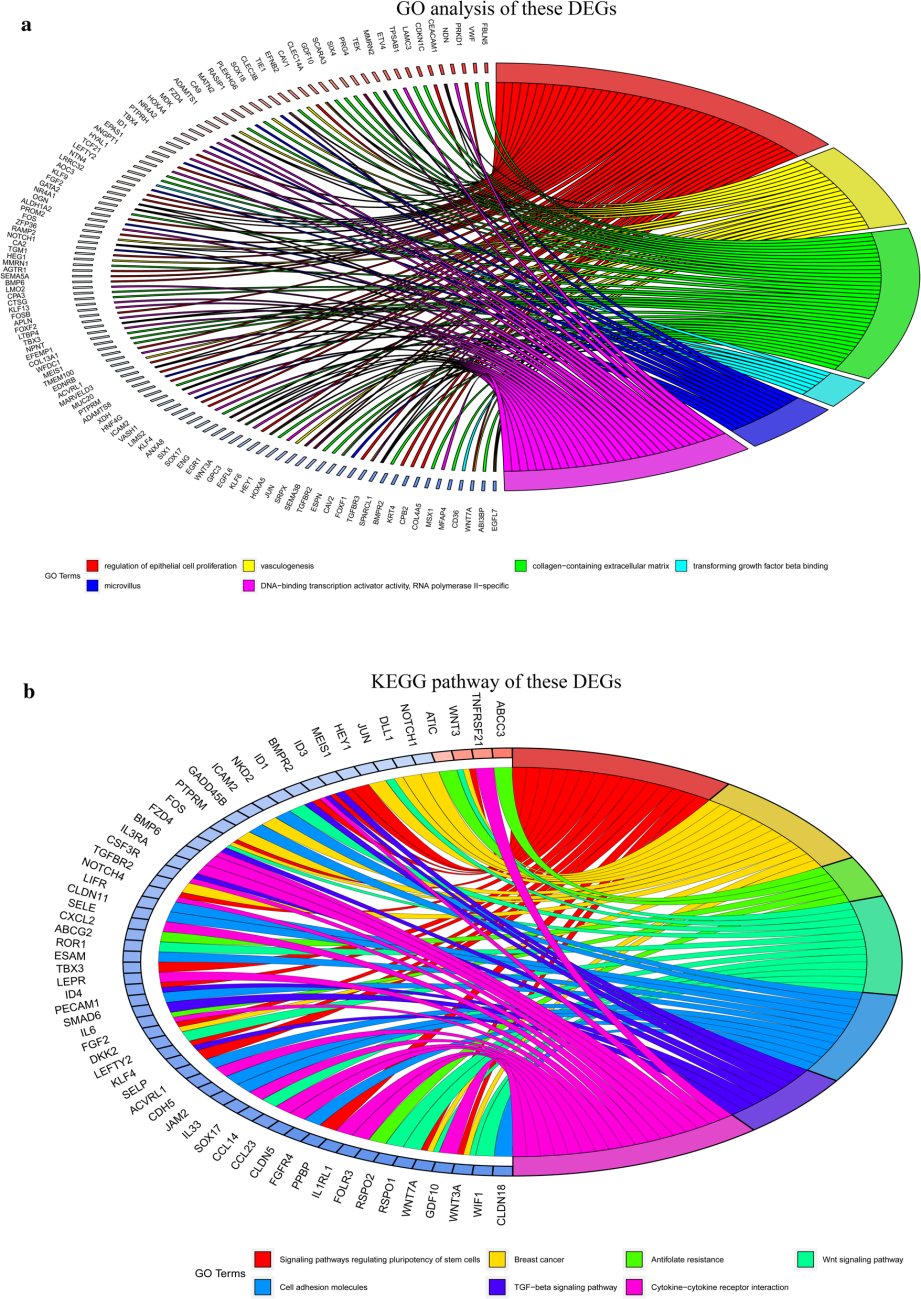
Functional enrichment analysis of differential co-expression genes using the clusterProfiler package. **a** Gene ontology (GO) enrichment analysis of differential co-expression genes. **b** Kyoto encyclopedia of genes and genomes pathway (KEGG) of differential co-expression genes

The outcomes of BP analysis of these genes showed that the regulation of epithelial cell proliferation and vasculogenesis were significantly enriched. CC analysis revealed that collagen-containing extracellular matrix and microvilli were associated with 486 genes. According to the outcomes of the MF analysis, transforming growth factor beta binding and DNA-binding transcription activator activity, RNA polymerase II-specific genes were primarily enriched (Fig. 5a). Furthermore, KEGG pathway results illustrated that signaling pathways regulating the pluripotency of stem cells, breast cancer and antifolate resistance were mainly enriched (Fig. 5b).

### PPI network construction and hub genes selection

**Fig. 6:**
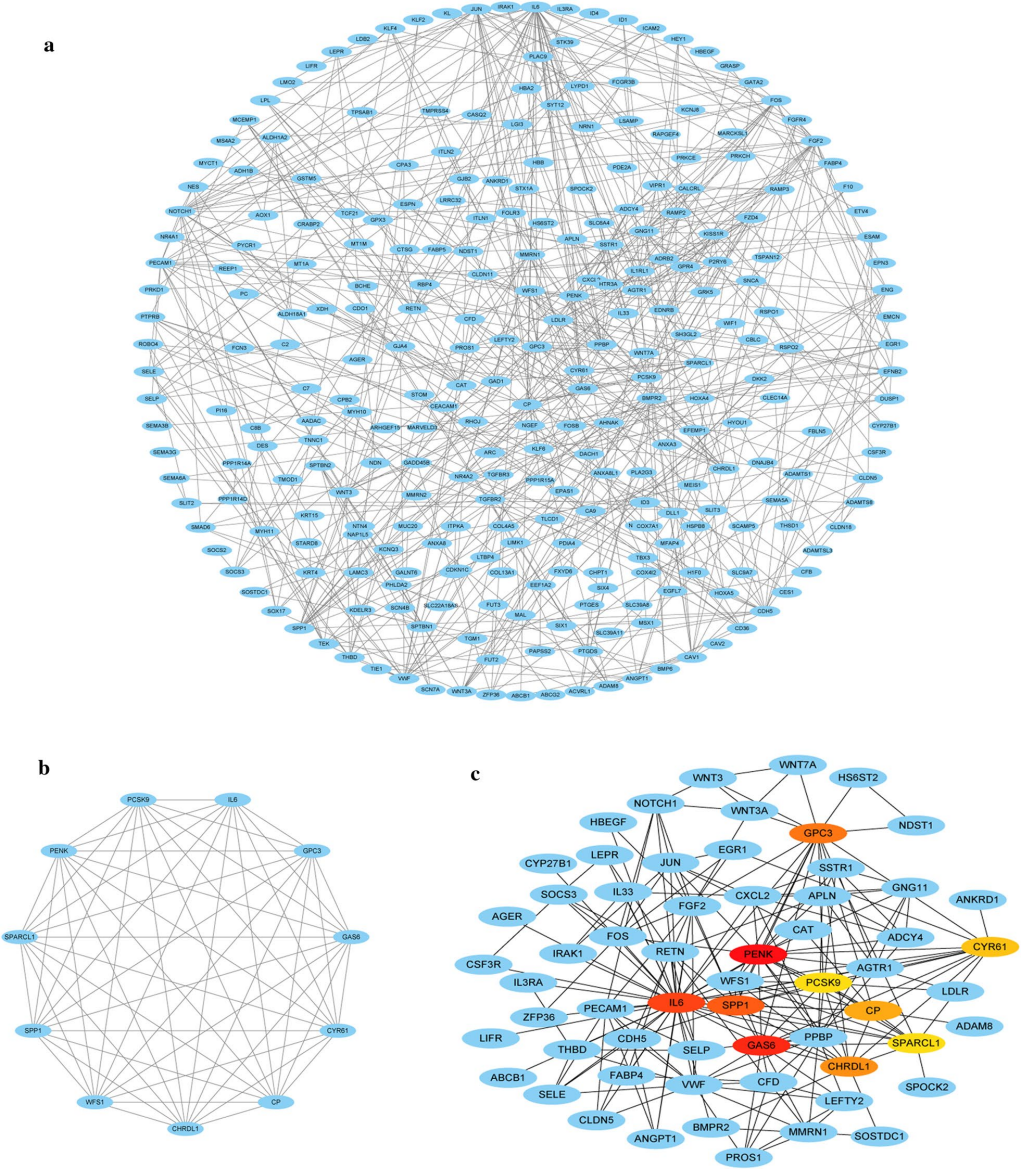
Visualization of the protein–protein interaction (PPI) network, the most significant module and hub genes. **a** PPI network of differential co-expression genes. **b** The most significant module from PPI network. **c** Selection of hub genes from PPI network through maximal clique centrality (MCC) algorithm. The turquoise nodes represent the genes. Edges suggest the protein–protein relations. The red nodes represent genes with high MCC values, while the yellow nodes represent genes with low MCC values

The PPI network of differentially coexpressed genes was displayed in Fig. 6a, which included 283 nodes and 632 edges. The most significant module was found using the MCODE plug-in, which contained 11 nodes and 55 edges (Fig. 6b). Then, hub genes were identified according to the rank of MCC values. The top 10 genes (PENK, GAS6, IL6, SPP1, GPC3, CHRDL1, CP, CYR61, WFS1 and SPARCL1) were recognized as hub genes. These hub genes selected from the PPI network are clearly illustrated in Fig. 6c, and the shade of the color represents the magnitude of the MCC scores.

### Prognostic roles of hub genes and relation with pathological stages

**Fig. 7:**
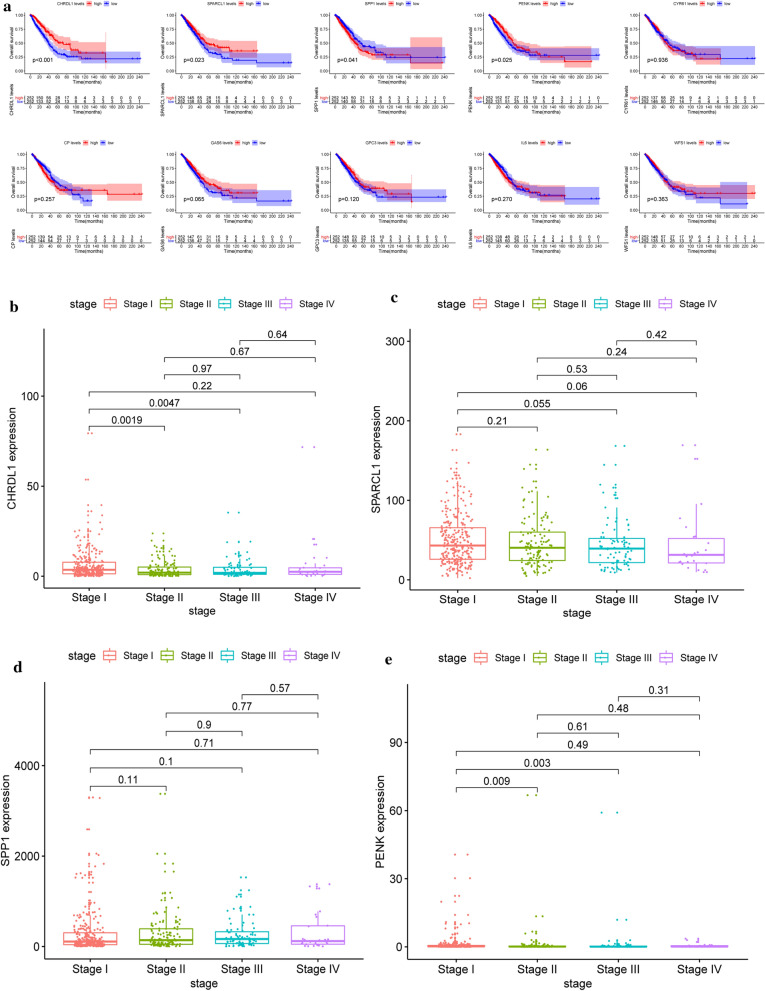
Prognostic roles of 10 hub genes and relation with pathological stages in patients of TCGA-LUAD dataset. Survival analysis for **a** CHRDL1, SPARCL1, SPP1, PENK, CYR61, CP, GAS6, GPC3, IL6 and WFS1 in LUAD. The LUAD patients are divided into high expression cohort (red) and low expression cohort (blue) according to the median expression of hub genes. Log-rank P < 0.05 is believed a statistical difference. **b** The relations of **b** CHRDL1, **c** SPARCL1, **d** SPP1 and **e** PENK with pathological stages among patients from TCGA-LUAD dataset

To explore the prognostic roles of the top 10 hub genes in LUAD, survival analysis was performed using the survival information of the TCGA-LUAD dataset. Lower expression of CHRDL1, SPARCL1 and PENK correlated with the poor prognosis among LUAD patients, while higher expression of SPP1 was correlated with poor prognosis (Fig. 7a). In addition, we also explored the relationship between the expression levels of hub genes and pathological stages (Fig. 7b–e).

### External validation of public databases

**Fig. 8:**
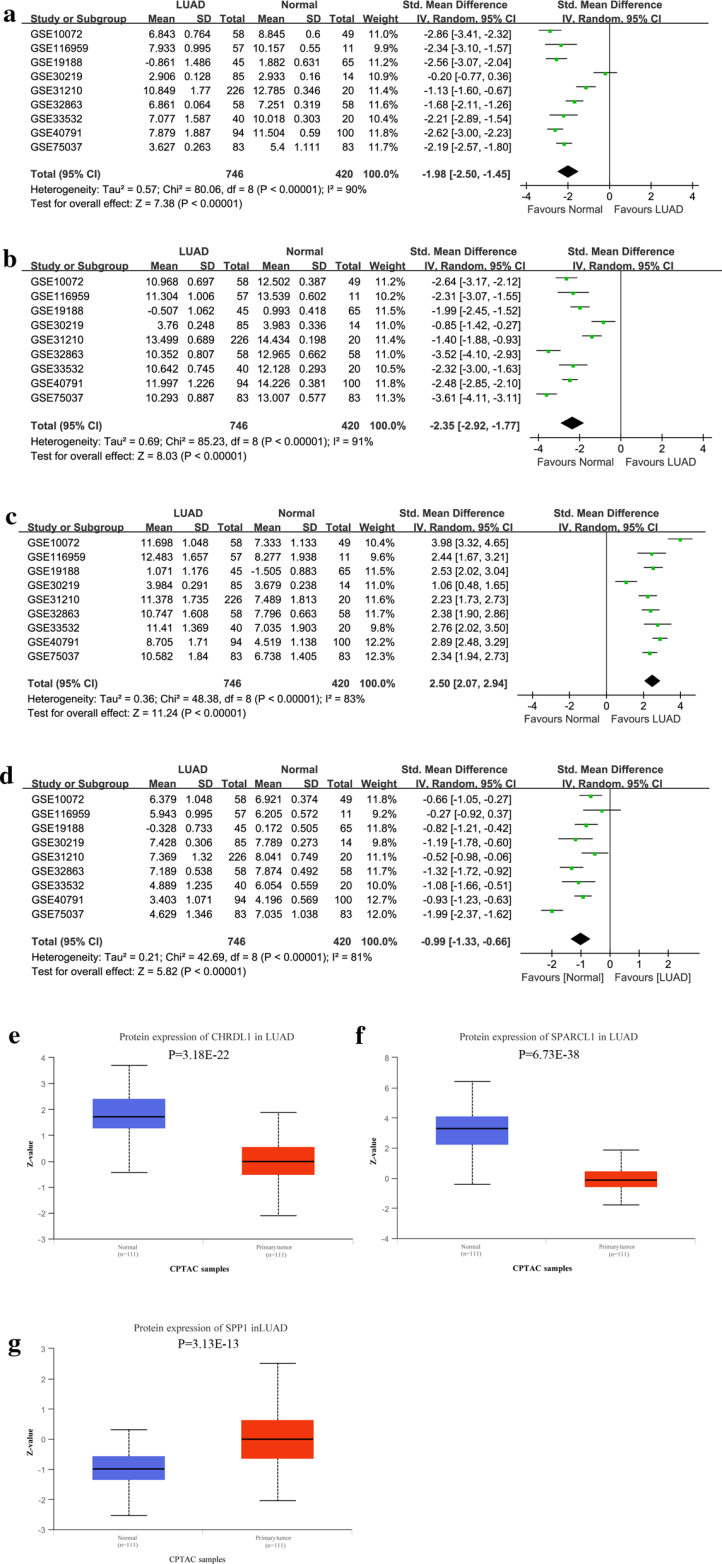
External validation of the expression patterns of survival-related hub genes based on GSE19188 and Clinical Proteomic Tumor Analysis Consortium (CPTAC) database. The mRNA (**a**) and protein (**b**) expression patterns of CHRDL1 are compared between LUAD and normal lung tissues. The mRNA (**c**) and protein (**d**) expression patterns of SPARCL1 are compared between LUAD and normal lung tissues. The mRNA (**e**) and protein (**f**) expression patterns of SPP1 are compared between LUAD and normal lung tissues. The mRNA (**e**) expression pattern of PENK is compared between LUAD and normal lung tissues

To increase the reliability of our findings, three external databases were used in our study. First, nine datasets satisfying the selection criteria were included for the comparisons of mRNA patterns of OS-related genes (Table 1). Comprehensive meta-analyses of the nine datasets indicated that CHRDL1 (Fig. 8a), SPARCL1 (Fig. 8b) and PENK (Fig. 8d) were downregulated in LUAD tissues, whereas SPP1 was upregulated (Fig. 8c). Second, we still compared the protein expression levels of survival-related genes in the CPTAC (Fig. 8e–g) and HPA (Additional file 1: Figure S1; Additional file 2: Figure S2) databases. Table 2 shows the detailed results of the IHC analysis of these genes based on the HPA database. Although the expression level of PENK was missing in the CPTAC database, the protein expression patterns of CHRDL1, SPARCL1, and SPP1 were consistent with their mRNA expression patterns.

**Table 1:** Characteristics of the included datasets from GEO

GEO datasets	Publication year	Country	RNA-Seq platforms	Normal	LUAD	Sum
GSE10072	2008	USA	GPL96	49	58	107
GSE116959	2019	France	GPL17077	11	57	68
GSE19188	2010	Netherlands	GPL570	65	45	110
GSE30219	2013	France	GPL570	14	85	99
GSE31210	2011	Japan	GPL570	20	226	246
GSE32863	2012	USA	GPL6884	58	58	116
GSE33532	2014	Germany	GPL570	20	40	60
GSE40791	2013	USA	GPL570	100	94	194
GSE75037	2016	USA	GPL6884	83	83	166

*GEO* Gene Expression Omnibus, *LUAD* lung adenocarcinoma

**Table 2:** The detailed information of immunohistochemistry from the Human Protein Atlas database

Gene	Normal lung tissues								LUAD tissues			
					Pneumocytes				Tumor cells			
	Staining	Intensity	Quantity	Location	Staining	Intensity	Quantity	Location	Staining	Intensity	Quantity	Location
CHRDL1	Medium	Moderate	75–25%	Cytoplasmic/membranous	Low	Weak	75–25%	Cytoplasmic/membranous	Not detected	Negative	None	None
SPARCL1	Not detected	Weak	< 25%	Cytoplasmic/membranous	Not detected	Negative	None	None	Not detected	Negative	None	None
SPP1	Low	Weak	75–25%	Cytoplasmic/membranous	Not detected	Negative	None	None	Not detected	Negative	None	None
PENK	Low	Weak	> 75%	Cytoplasmic/membranous	Not detected	Negative	None	None	Not detected	Negative	None	None

*LUAD* lung adenocarcinoma

### GSEA of survival-related hub genes

**Fig. 9:**
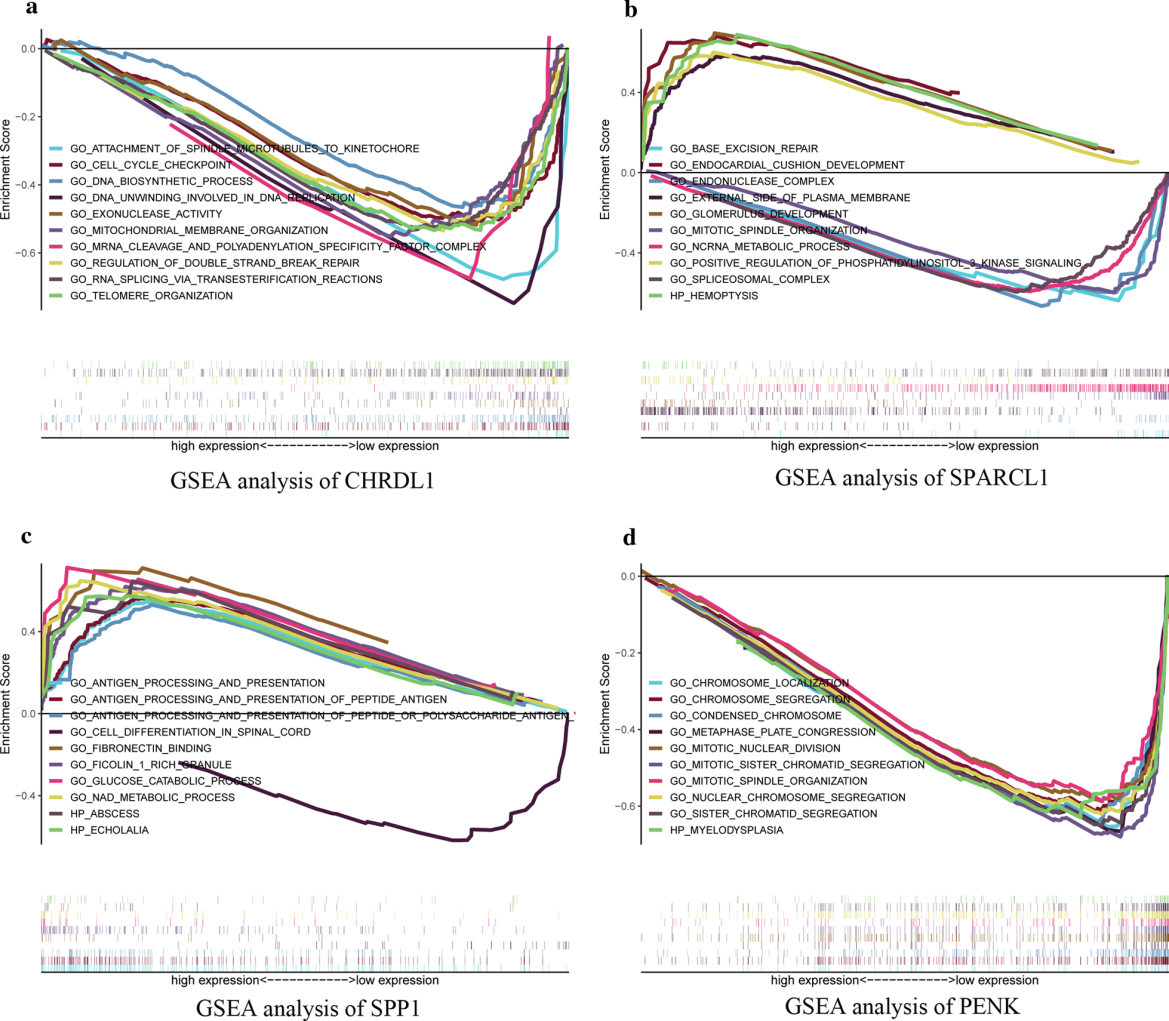
Enrichment plots by gene set enrichment analysis (GSEA). Relative pathways associated with the expression of CHRDL1 (**a**), SPARCL1 (**b**), SPP1 (**c**), and PENK (**d**) are illustrated

GSEA showed that DNA-dependent DNA replication, mitotic metaphase plate congression, and mitotic sister chromatid segregation were associated with CHRDL1 (Fig. 9a). In addition, GSEA suggested that catalytic activity acting on RNA, DNA packing and mesenchymal morphogenesis were correlated with SPARCL1 (Fig. 9b). Their detailed outcomes of GSEA are displayed in Table 3. Glucose catabolic process and antigen procession and presentation were correlated with SPP1 (Fig. 9c), while mitotic sister chromatid segregation and mitotic nuclear division were associated with CHRDL1 (Fig. 9d). And their detailed results of GSEA are demonstrated in Additional file 10: Table S8.

**Table 3:** Relative pathways associated with the expression of CHRDL1 and SPARCL1 using GSEA

Gene	Name	ES	NES	NOM p-value	FDR q-value
CHRDL1	GO_REGULATION_OF_DOUBLE_STRAND_BREAK_REPAIR	− 0.55	− 1.93	< 0.0001	0.109
	GO_CELL_CYCLE_CHECKPOINT	− 0.51	− 1.93	0.006	0.107
	GO_DNA_UNWINDING_INVOLVED_IN_DNA_REPLICATION	− 0.81	− 1.93	< 0.0001	0.106
	GO_EXONUCLEASE_ACTIVITY	− 0.53	− 1.92	0.002	0.116
	GO_RNA_SPLICING_VIA_TRANSESTERIFICATION_REACTIONS	− 0.53	− 1.92	0.006	0.114
	GO_TELOMERE_ORGANIZATION	− 0.54	− 1.91	0.002	0.114
	GO_ATTACHMENT_OF_SPINDLE_MICROTUBULES_TO_KINETOCHORE	− 0.71	− 1.91	< 0.0001	0.114
	GO_MITOCHONDRIAL_MEMBRANE_ORGANIZATION	− 0.56	− 1.91	0.002	0.113
	GO_MRNA_CLEAVAGE_AND_POLYADENYLATION_SPECIFICITY_FACTOR_COMPLEX	− 0.73	− 1.91	0.002	0.112
	GO_DNA_BIOSYNTHETIC_PROCESS	− 0.47	− 1.91	0.002	0.112
SPARCL1	GO_ENDONUCLEASE_COMPLEX	− 0.68	− 2.17	< 0.0001	0.047
	GO_BASE_EXCISION_REPAIR	− 0.67	− 2.11	0.002	0.048
	GO_SPLICEOSOMAL_COMPLEX	− 0.6	− 2.1	< 0.0001	0.031
	GO_NCRNA_METABOLIC_PROCESS	− 0.59	− 2.1	< 0.0001	0.048
	GO_MITOTIC_SPINDLE_ORGANIZATION	− 0.61	− 2.09	< 0.0001	0.048
	GO_GLOMERULUS_DEVELOPMENT	0.69	2.27	< 0.0001	0.046
	HP_HEMOPTYSIS	0.69	2.17	< 0.0001	0.033
	GO_EXTERNAL_SIDE_OF_PLASMA_MEMBRANE	0.58	2.16	< 0.0001	0.048
	GO_ENDOCARDIAL_CUSHION_DEVELOPMENT	0.68	2.16	< 0.0001	0.047
	GO_POSITIVE_REGULATION_OF_PHOSPHATIDYLINOSITOL_3_KINASE_SIGNALING	0.6	2.16	< 0.0001	0.047

*GSEA* gene set enrichment analysis, *NES* normalized enrichment score, *NOM* nominal, *FDR* false discovery rate

### Immunohistochemical verification

**Fig. 10:**
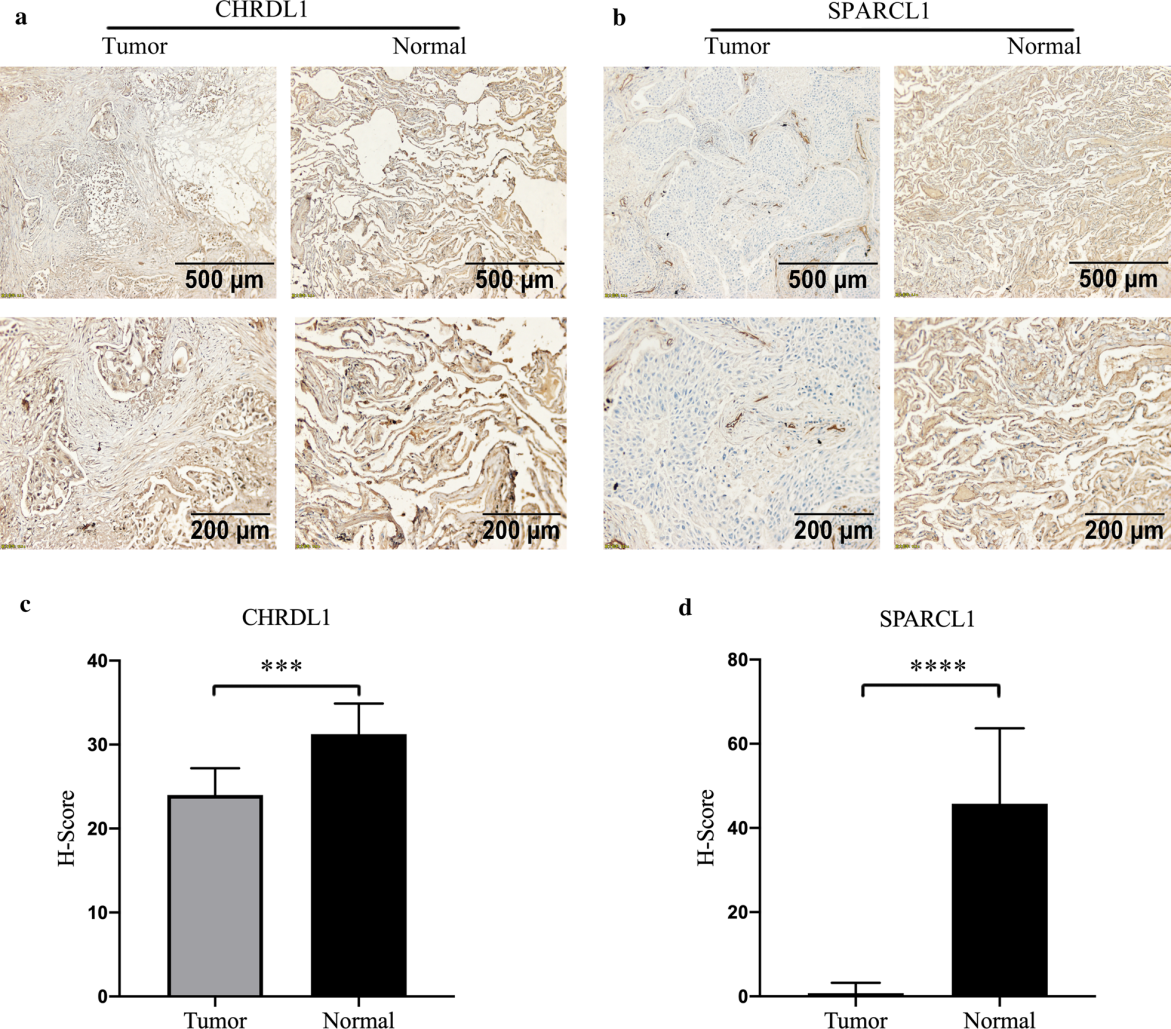
The distribution and expression of CHRDL1 and SPARCL1 proteins in twenty pairs of LUAD and normal tissues. Representative pictures of immunohistochemistry of CHRDL1 and SPARCL1 proteins are shown (**a, b**). The score of immunohistochemistry of CHRDL1 and SPARCL1 proteins are displayed (**c, d**). ***P = 0.001; ****P < 0.001

To increase the reliability of our findings, we investigated the distribution and expression of CHRDL1 and SPARCL1 proteins in five pairs of randomly selected tissues. Representative IHC images revealed that CHRDL1 and SPARCL1 proteins were primarily distributed in the cytoplasm, partially on the cell membrane (Fig. 10a, b). Furthermore, both were obviously downregulated in LUAD tissues (Fig. 10c, d), which were consistent with our previous results.

## Discussion

As a prevalent cancer associated with high mortality, lung cancer has resulted in substantial socioeconomic burdens to lung cancer patients and countries. Progress in LUAD therapy has been made in recent years, but the diagnosis and prognosis of LUAD remain poor because of the lack of precise molecular biomarkers. Thus, better indicators for the specific prognosis and progression of patients with LUAD are urgently required. In our analysis, a list of 486 differentially coexpressed genes was selected using the GSE75037 and TCGA-LUAD datasets through comprehensive bioinformatics analysis. These genes were significantly enriched in the regulation of epithelial cell proliferation, collagen-containing extracellular matrix, transforming growth factor beta binding and signaling pathways regulating pluripotency of stem cells. According to the rank of MCC scores, the top ten genes were identified as hub genes related to LUAD. Then, we found that 4 hub genes (namely, CHRDL1, SPARCL1, PENK and SPP1) of the top 10 genes were closely correlated with OS among patients with LUAD, and CHRDL1, SPARCL1 and PENK were positively correlated with the survival of LUAD, while SPP1 was negatively correlated with the prognosis. Based on external validation of the GEO, CPTAC, and THPA databases, we observed that the mRNA and protein expression levels of CHRDL1, SPARCL1 and PENK were lower in LUAD, while SPP1 was upregulated. GSEA showed that DNA-dependent DNA replication and catalytic activity acting on RNA were associated with the expression of CHRDL1 and SPARCL1, respectively. Finally, the IHC outcomes validated the expression status of CHRDL1 and SPARCL1 in LUAD.

CHRDL1, namely, Chordin Like 1, is a specific antagonist of bone morphogenetic protein (BMP), and BMP signaling participates in many responses, including cell proliferation, migration and invasion in various cancers [20]. CHRDL1 was observed to be notably downregulated in many cancers [21]. Pei et al. observed that the CHRDL1 promoter was hypermethylated in gastric cancer, which may explain the downregulation of CHRDL1 in gastric cancer. Additionally, low expression of CHRDL1 was associated with worse survival among 100 patients with gastric cancer. In addition, these authors reported that the knockdown of CHRDL1 induced cell proliferation and metastasis via the activation of Akt and Erk, suggesting that CHRDL1 plays a tumor suppressor role in gastric cancer [22]. Wang et al. suggested that miRNA hsa‐mir‐204 contributed to cell proliferation, migration and invasion through the downregulation of CHRDL1 in gastric cancer [23]. Moreover, CHRDL1 could inhibit cell migration and invasion by suppressing BMP signaling in breast cancer [24]. CHRDL1 was found to be less expressed in thyroid cancer using the IHC method, and CHRDL1 was closely correlated with disease-free survival (DFS) of patients with thyroid cancer [25]. However, the role of CHRDL1 in LUAD has not been reported before our study, so additional studies exploring the role of CHRDL1 in LUAD are needed to further confirm our findings.

The secreted protein acidic rich in cysteine-like 1 (SPARCL1) is a matricellular protein that belongs to the SPARC-related protein family. SPARCL1 inhibited the progression of tumor cells from the G1 phase to the S phase and participated in the negative regulation of cell proliferation [26]. Many studies have revealed the downregulated status and role of SPARCL1 in various cancers [27]. For example, SPARCL1 inhibited cell proliferation and invasion by inhibiting the mitogen-activated protein kinase kinase (MEK) and extracellular signal-related kinase (ERK) pathways in ovarian cancer [28]. Moreover, miR-539-3p was found to promote cell invasion by targeting SPARCL1 in epithelial ovarian cancer [29]. SPARCL1 was downregulated in gastrointestinal stromal tumors, which contributed to cell migration and invasion, and SPARCL1 can predict the prognosis of gastrointestinal stromal tumors (P = 0.008) [30]. Similarly, higher expression of SPARCL1 was correlated with better cell differentiation and less distant metastasis in colorectal cancers than those with lower expression of SPARCL1, and SPARCL1 was positively correlated with the prognosis of colorectal cancers (P < 0.01) [31]. Wang et al. observed that SPARCL1 was a DNA methylation-regulated gene, and this gene was downregulated in LUAD [32]. Considering these reports and our findings, we can conclude that SPARCL1 and CHRDL1 play therapeutic and prognostic roles in the carcinogenesis and metastasis of LUAD.

Admittedly, several limitations existed in this analysis. (1) Integrated bioinformatics analysis was performed in our study to identify candidate prognostic genes in LUAD, but it might not be highly accurate for patients with each LUAD subtype. (2) Although the GSE75037 and TCGA-LUAD datasets had many LUAD samples, only the two datasets were included in our analysis. (3) We did not validate our findings by conducting further experiments in addition to IHC, and more relevant basic experiments are required for further validation.

## Conclusion

In general, this analysis was performed to find hub genes that might be correlated with the initiation and progression of LUAD using differential gene expression analysis and WGCNA. Ten hub genes were identified according to the rank of MCC values, and four genes were significantly associated with OS. Furthermore, CHRDL1 and SPARCL1 were candidate therapeutic and prognostic biomarkers of LUAD.

## Supplementary Information

**Additional file 1: Figure S1.** External validation of the expression levels of survival-related hub genes based on the Human Protein Atlas (THPA) database. The immunohistochemistry (IHC) results of CHRDL1 (A) and SPARCL1 (B) are compared between LUAD and normal lung tissues.

**Additional file 2: Figure S2.** External validation of the expression levels of survival-related hub genes based on the Human Protein Atlas (THPA) database. SPP1 (A), and PENK (B) are compared between LUAD and normal lung tissues.

**Additional file 3: Table S1.** The search strategy and selection criteria.

**Additional file 4: Table S2.** Major demographic and clinicopathological characteristics of patients with LUAD.

**Additional file 5: Table S3.** The differentially expressed genes (DEGs) in GSE75037.

**Additional file 6: Table S4.** The differentially expressed genes (DEGs) in TCGA-LUAD dataset.

**Additional file 7: Table S5.** The co-expression genes in the blue module of GSE75037.

**Additional file 8: Table S6.** The co-expression genes in the blue module of TCGA-LUAD dataset.

**Additional file 9: Table S7.** The intersection of genes among the two lists of differentially expressed genes (DEGs) and the two lists of co-expression genes.

**Additional file 10: Table S8.** Relative pathways associated with the expression of SPP1 and PENK using GSEA.

## Data Availability

The datasets downloaded and analyzed during the current study are available on the TCGA and GEO database: TCGA-LUAD database: https://portal.gdc.cancer.gov/; GSE75037: https://www.ncbi.nlm.nih.gov/geo/query/acc.cgi?acc=GSE75037; GSE19188: https://www.ncbi.nlm.nih.gov/geo/query/acc.cgi?acc=GSE19188.

## Abbreviations

NSCLC: Non-small cell lung cancer
LUAD: Lung adenocarcinoma
WGCNA: Weighed Gene Co-expression Network Analysis
DEGs: Differentially expressed genes
GEO: Gene Expression Omnibus
TCGA: The Cancer Genome Atlas
CPTAC: Clinical Proteomic Tumor Analysis Consortium
THPA: The Human Protein Atlas
FDR: False discovery rate
TOM: Topological overlap matrix
FPKM: Fragments per kilobase per million
TPM: Transcript per million
MCC: Maximal clique centrality
IHC: Immunohistochemistry
NES: Normalized enrichment score
GO: Gene ontology
KEGG: Kyoto encyclopedia of genes and genomes pathway
PPI: Protein–protein interaction network
STRING: Search Tool for the Retrieval of Interacting Genes
MCODE: Molecular Complex Detection
BP: Biological processes
CC: Cellular component
MF: Molecular function
OS: Overall survival
HR: Hazard ratio
GSEA: Gene set enrichment analysis
BMP: Bone morphogenetic protein
DFS: Disease-free survival
MEK: Mitogen-activated protein kinase kinase
ERK: Extracellular signal-related kinase
