# Supplementary figures and images for: Low expression of CHRDL1 and SPARCL1 predicts poor prognosis of lung adenocarcinoma based on comprehensive analysis and immunohistochemical validation
Source: Cancer Cell Int. 2021 May 12;21:259. doi: 10.1186/s12935-021-01933-9 (PMC8117659; doi:10.1186/s12935-021-01933-9)

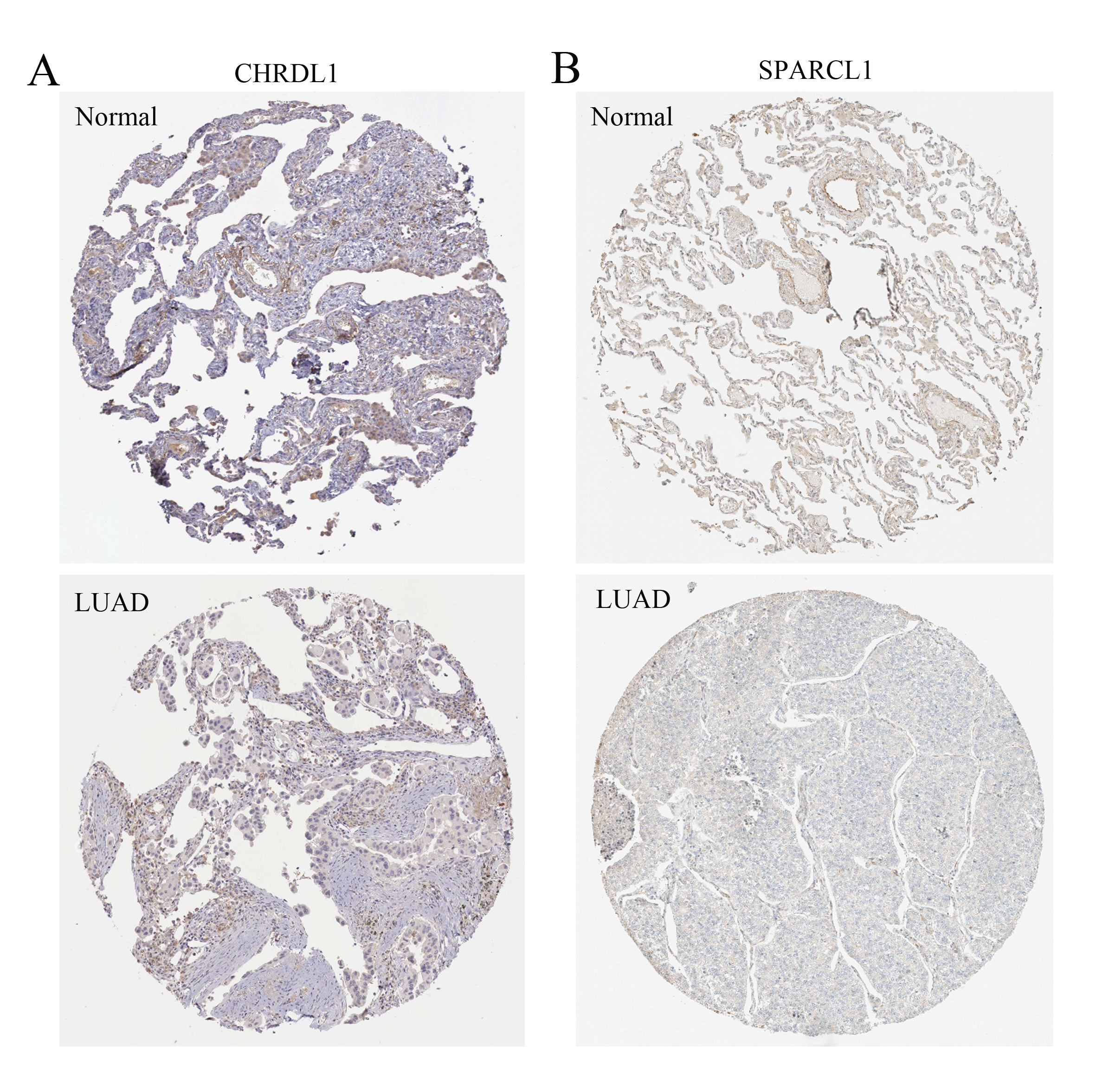

Supplement: Supplementary file 1 — Additional file 1: Figure S1. External validation of the expression levels of survival-related hub genes based on the Human Protein Atlas (THPA) database. The immunohistochemistry (IHC) results of CHRDL1 (A) and SPARCL1 (B) are compared between LUAD and normal lung tissues. [file 12935_2021_1933_MOESM1_ESM.tif]

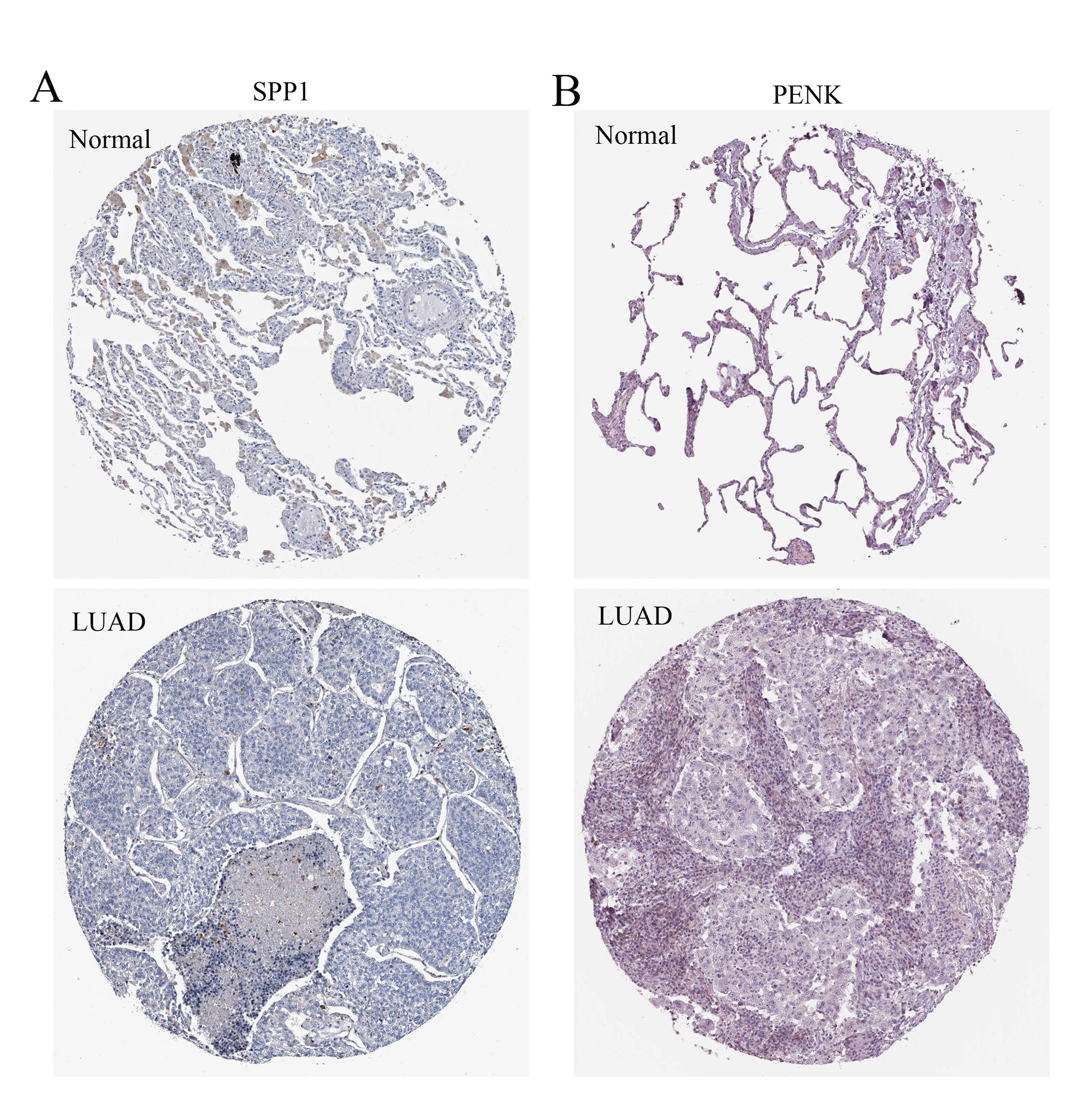

Supplement: Supplementary file 2 — Additional file 2: Figure S2. External validation of the expression levels of survival-related hub genes based on the Human Protein Atlas (THPA) database. SPP1 (A), and PENK (B) are compared between LUAD and normal lung tissues. [file 12935_2021_1933_MOESM2_ESM.tif]
