# Supplementary material for: Low expression of CHRDL1 and SPARCL1 predicts poor prognosis of lung adenocarcinoma based on comprehensive analysis and immunohistochemical validation
Source: Cancer Cell Int. 2021 May 12;21:259. doi: 10.1186/s12935-021-01933-9 (PMC8117659; doi:10.1186/s12935-021-01933-9)
Supplement: Supplementary file 3 — Additional file 3: Table S1. The search strategy and selection criteria. [file 12935_2021_1933_MOESM3_ESM.docx]

**Table S1** The search strategy and selection criteria

*The search strategy in GEO database*

(((((((((((lung cancer) OR lung adenocarcinoma) OR LUAD) OR lung tumor) OR lung carcinoma) OR lung neoplasm) OR pulmonary adenocarcinoma) OR pulmonary cancer) OR pulmonary tumor) OR pulmonary carcinoma) OR pulmonary neoplasm).

*Selection criteria for included datasets from GEO*

(1) sample sizes were large enough (the number of LUAD samples≥40 and total number of LUAD and normal lung samples≥60); (2) the expression profiles of LUAD and non-malignant lung tissues were provided simultaneously; and (3) the organisms were Homo sapiens.
