# Supplementary material for: Low expression of CHRDL1 and SPARCL1 predicts poor prognosis of lung adenocarcinoma based on comprehensive analysis and immunohistochemical validation
Source: Cancer Cell Int. 2021 May 12;21:259. doi: 10.1186/s12935-021-01933-9 (PMC8117659; doi:10.1186/s12935-021-01933-9)
Supplement: Supplementary file 4 — Additional file 4: Table S2. Major demographic and clinicopathological characteristics of patients with LUAD. [file 12935_2021_1933_MOESM4_ESM.docx]

**Table S2** Major demographic and clinicopathological characteristics of patients with LUAD.

| Baseline characteristics | Value^*^ |
| --- | --- |
| **Age (years)** | 62.5 (39-74) |
| **Gender** |  |
| Male | 11 (55) |
| Female | 9 (45) |
| **Smoking status** |  |
| Yes | 9 (45) |
| No | 11 (55) |
| **Tumor size (cm)** |  |
| <5 | 18 (90) |
| >5 | 2 (10) |
| **Grade** |  |
| Low | 6 (30) |
| Moderate-low | 12 (60) |
| Moderate | 2 (10) |
| **Lymph node metastasis** |  |
| Yes | 12 (60) |
| No | 8 (40) |
| **Stage** |  |
| I | 4 (20) |
| II | 10 (50) |
| III | 6 (30) |
| **Tumor recurrence or metastasis** |  |
| Yes | 17 (85) |
| No | 3 (15) |

**Abbreviations:** LUAD: lung adenocarcinoma.* values are expressed as median (range) or n (%).
