# Supplementary material for: Low expression of CHRDL1 and SPARCL1 predicts poor prognosis of lung adenocarcinoma based on comprehensive analysis and immunohistochemical validation
Source: Cancer Cell Int. 2021 May 12;21:259. doi: 10.1186/s12935-021-01933-9 (PMC8117659; doi:10.1186/s12935-021-01933-9)
Supplement: Supplementary file 10 — Additional file 10: Table S8. Relative pathways associated with the expression of SPP1 and PENK using GSEA. [file 12935_2021_1933_MOESM10_ESM.docx]

**Table S8** Relative pathways associated with the expression of SPP1 and PENK using GSEA.

| **Gene** | **Name** | **ES** | **NES** | **NOM**  **p -value** | **FDR**  **q-value** |
| --- | --- | --- | --- | --- | --- |
| SPP1 | GO_FICOLIN_1_RICH_GRANULE | 0.62 | 2.14 | <0.0001 | 0.221 |
|  | GO_NAD_METABOLIC_PROCESS | 0.65 | 2.12 | 0.002 | 0.154 |
|  | GO_GLUCOSE_CATABOLIC_PROCESS | 0.71 | 2.10 | <0.0001 | 0.129 |
|  | GO_ANTIGEN_PROCESSING_AND_PRESENTATION | 0.55 | 2.06 | <0.0001 | 0.217 |
|  | GO_ANTIGEN_PROCESSING_AND_PRESENTATION_OF_PEPTIDE_ANTIGEN | 0.57 | 2.05 | <0.0001 | 0.208 |
|  | GO_CELL_DIFFERENTIATION_IN_SPINAL_CORD | -0.65 | -2.13 | <0.0001 | 0.165 |
|  | GO_ANTIGEN_PROCESSING_AND_PRESENTATION_OF_PEPTIDE_OR_  POLYSACCHARIDE_ANTIGEN_VIA_MHC_CLASS_II | 0.53 | 1.99 | <0.0001 | 0.237 |
|  | HP_ECHOLALIA | 0.57 | 1.99 | 0.002 | 0.224 |
|  | GO_FIBRONECTIN_BINDING | 0.71 | 1.98 | 0.004 | 0.218 |
|  | HP_ABSCESS | 0.65 | 1.98 | <0.0001 | 0.211 |
|  |  |  |  |  |  |
| PENK | GO_MITOTIC_SISTER_CHROMATID_SEGREGATION | -0.69 | -2.19 | <0.0001 | 0.065 |
|  | GO_SISTER_CHROMATID_SEGREGATION | -0.66 | -2.14 | <0.0001 | 0.077 |
|  | HP_MYELODYSPLASIA | -0.64 | -2.14 | <0.0001 | 0.053 |
|  | GO_CHROMOSOME_LOCALIZATION | -0.67 | -2.13 | <0.0001 | 0.054 |
|  | GO_MITOTIC_NUCLEAR_DIVISION | -0.59 | -2.13 | <0.0001 | 0.045 |
|  | GO_METAPHASE_PLATE_CONGRESSION | -0.69 | -2.11 | <0.0001 | 0.050 |
|  | GO_CHROMOSOME_SEGREGATION | -0.62 | -2.11 | <0.0001 | 0.046 |
|  | GO_NUCLEAR_CHROMOSOME_SEGREGATION | -0.62 | -2.10 | <0.0001 | 0.048 |
|  | GO_CONDENSED_CHROMOSOME | -0.65 | -2.10 | <0.0001 | 0.044 |
|  | GO_MITOTIC_SPINDLE_ORGANIZATION | -0.60 | -2.08 | <0.0001 | 0.056 |

**Abbreviations:** GSEA: Gene Set Enrichment Analysis; NES: normalized enrichment score; NOM: nominal; FDR: false discovery rate.
